# A comparison of results of empirical studies of supplementary search techniques and recommendations in review methodology handbooks: a methodological review

**DOI:** 10.1186/s13643-017-0625-1

**Published:** 2017-11-28

**Authors:** Chris Cooper, Andrew Booth, Nicky Britten, Ruth Garside

**Affiliations:** 1 PenTAG, University of Exeter Medical School, Exeter, England; 2 HEDS, School of Health and Related Research (ScHARR), University of Sheffield, Sheffield, England; 3 Institute of Health Research, University of Exeter Medical School, Exeter, England; 4 European Centre for Environment and Human Health, University of Exeter Medical School, Truro, England

**Keywords:** Supplementary searching, Systematic reviews, Handsearching, Citation searching, Web searching, Trial searching, Author contact, Handbooks, Information science

## Abstract

**Background:**

The purpose and contribution of supplementary search methods in systematic reviews is increasingly acknowledged. Numerous studies have demonstrated their potential in identifying studies or study data that would have been missed by bibliographic database searching alone.

What is less certain is how supplementary search methods actually work, how they are applied, and the consequent advantages, disadvantages and resource implications of each search method.

The aim of this study is to compare current practice in using supplementary search methods with methodological guidance.

**Methods:**

Four methodological handbooks in informing systematic review practice in the UK were read and audited to establish current methodological guidance.

Studies evaluating the use of supplementary search methods were identified by searching five bibliographic databases. Studies were included if they (1) reported practical application of a supplementary search method (descriptive) or (2) examined the utility of a supplementary search method (analytical) or (3) identified/explored factors that impact on the utility of a supplementary method, when applied in practice.

**Results:**

Thirty-five studies were included in this review in addition to the four methodological handbooks. Studies were published between 1989 and 2016, and dates of publication of the handbooks ranged from 1994 to 2014.

Five supplementary search methods were reviewed: contacting study authors, citation chasing, handsearching, searching trial registers and web searching.

**Conclusions:**

There is reasonable consistency between recommended best practice (handbooks) and current practice (methodological studies) as it relates to the application of supplementary search methods.

The methodological studies provide useful information on the effectiveness of the supplementary search methods, often seeking to evaluate aspects of the method to improve effectiveness or efficiency. In this way, the studies advance the understanding of the supplementary search methods. Further research is required, however, so that a rational choice can be made about which supplementary search strategies should be used, and when.

## Background

The purpose and contribution of supplementary search methods in systematic reviews are increasingly acknowledged. Numerous studies have demonstrated their potential in identifying studies or study data that would have been missed by bibliographic database searching alone [1–8].

It is commonly believed that the inclusion of supplementary search methods adds value to the process of comprehensive study identification in systematic reviews. The methodological handbooks for systematic review methodology, such as The Cochrane or CRD Handbooks, provide practical (although limited) instruction on how to undertake each supplementary search method, and empirical studies have evaluated the effectiveness and efficiencies of these search methods. What is perhaps less certain is how supplementary search methods actually work, and what the advantages, disadvantages and resource implications of each search method are.

### Study aim

The aim of this study is to compare empirical studies of supplementary search techniques to the recommendations in methodological handbooks.

By re-considering the best practice guidance of methodological handbooks for systematic review, and reviewing how this guidance has been interpreted and evaluated within current practice by authors, this study seeks to identify claimed advantages, claimed disadvantages and resource requirements of using supplementary search methods.

### The research question for this study

The research question for this study is how do empirical studies of supplementary search techniques compare to the recommendations in review methodology handbooks?

## Methods

This study aims to produce a structured methodological overview of methodological handbooks on the conduct of supplementary searches in systematic reviews. In addition, we reviewed studies that report on the utility and practice of supplementary searches. In order to identify this literature, a systematic approach to study identification, study selection and data extraction was used, which is set out below. These two types of literature—handbooks and practical explorations of applying supplementary search strategies—were then compared. The advantages, disadvantages and resource requirements of each method were evaluated.

### Study identification

We selected the following methodological handbooks as the most influential handbooks in informing systematic review practice in the UK. The current editions of each handbook were read and audited to establish current methodological guidance:

- The Cochrane Handbook for Systematic Reviews of Interventions (version 5.10, March 2011) [9];
- Systematic Reviews: CRD’s guidance for undertaking review in health care (2009) [10];
- The Campbell Information Retrieval Methods Group guide to information retrieval (October 2009) [11]; and
- The NICE manual to developing NICE guidelines (October 2014) [12].

The following five search methods, supplementary to database searches, were identified from these handbooks:

1. Contacting study authors or experts
2. Citation chasing
3. Handsearching
4. Trial register searching
5. Web searching.

In order to compare the existing handbook guidance to current practice, we identified studies that describe and/or evaluate how these methods are applied in practice. Studies were identified by searching five bibliographic databases: MEDLINE, EMBASE, LISTA, ASSIA and Web of Science in July 2016. Forward citation chasing was applied to studies meeting inclusion at full text, and the bibliographies were appraised. Tables of included studies were examined if aggregated within systematic reviews. The search syntax for bibliographic database searching is included as a supplementary file.

### Study selection

Studies were downloaded into Endnote X6 where manual de-duplication was performed. Studies were single screened by CC using the inclusion criteria below:

#### Inclusion criteria

For inclusion in this review, a study was required to:

(i) Report practical application of a supplementary search method (descriptive)
(ii) Examine the utility of a supplementary search method (analytical)
(iii) Identify/explore factors that impact on the utility of a supplementary method when applied in practice

#### Exclusion criteria

The following studies were excluded:

i) Studies reporting the use of supplementary search methods but not discussing the practical application of the method (such as listing their use to identify studies in a systematic review, i.e. ‘we handsearched the following journals’)
ii) Studies reported as abstracts or on-going studies
iii) Systematic reviews or reviews, in which case tables of included studies were examined to identify eligible primary studies

#### Data extraction

The following data were extracted: citation details, study design, claimed advantages, claimed disadvantages and resource requirements.

## Results

Thirty-five studies were included in this review in addition to the four methodological handbooks. Studies were published between 1989 and 2016, and handbooks were published between 1994 and 2014. Table 1 summarises which studies cited which handbooks as their source of methodological reference. The handbooks audited for this study cited only three studies: Eysenbach et al. (2001) was cited in The Cochrane Handbook, Hetherington et al. (1989) was cited in The Cochrane Handbook and The Campbell Handbook and Papaioannou et al. (2010) was cited in The Campbell Handbook (Table 1).

**Table 1:** Studies citing handbooks: handbooks citing studies

Study	Cochrane (1994) [6]	CRD (2008) [7]	Campbell (2010) [8]	NICE Handbook (2013) [9]	Other source
Adams et al. [25]	X	X	X	X	NR
Armstrong et al. [31]	✓	X	X	X	X
Bakkalbasi et al. [16]	X	X	X	X	NR
Blumle and Antes [32]	✓	X	X	X	X
Bramer et al. [24]	✓	X	X	X	X
Briscoe [41]	✓	✓	X	✓	X
Croft [33]	✓	X	X	X	X
*Eysenbach et al.* [45]	X**	X	X	X	NR
Falagas et al. [23]	X	X	X	X	NR
Gibson et al. [14]	X	X	X	X	NR
Glanville et al. [34]	✓	X	X	X	X
Glanville et al. [38]	X	X	X	X	NR
Godin et al. [43]	✓	X	X	X	X
Hay et al. [26]	X	X	X	X	NR
*Hetherington et al.* [17]	X**	X	X**	X	Study predates any handbook
Hinde et al. [2]	✓	X	X	✓	X
Hopewell et al. [27]	X	X	X	X	NR
Jadad et al. [28]	X	X	X	X	Study predates any handbook
Janssens et al. [20]	✓	X	X	X	NR
Jones et al. [40]	✓	✓	X	X	Institute of Medicine
Langham et al. [30]	X	X	X	X	NR
Levay et al. [1]	✓	X	X	✓	X
Mahood at al. [44]	✓	X	✓	X	X
Mattioli et al. [35]	X	X	X	X	NR
McManus et al. [6]	X	X	X	X	NR
Milne and Thorogood [36]	X	X	X	X	NR
Moher [39]	X	X	X	X	NR
O’Leary [15]	X	X	X	X	NR
*Papaioannouet al.* [3]	✓	X	X**	X	X
Reveiz et al.[18]	X	X	X	X	
Robinson et al. [21]	X	X	X	X	NR
Selph et al. [12]	✓	X	X	X	Methods Guide for Effectiveness and Comparative Effectiveness Reviews
Stansfield et al. [42]	✓	X	✓	X	Collaboration for Environmental Evidence
Van Enst et al. [39]	✓	X	X	X	X
Wright et al. [22]	✓	✓	✓	X	Institute of Medicine
TOTAL	17	3	3	3	3 other sources

Studies in italics are ones cited by the handbooks identified using the symbol ‘**’ for informing their guidance

*NR* not reported

The results were categorised by the supplementary search methods and reported in five domains: (1) what the method is used for, (2) what the evidence says, (3) claimed advantages, (4) claimed disadvantages and (5) resource requirements. A summary of these results is presented in Table 2.

**Table 2:** Overview of results

Method	Includes	What is the method used for	What the evidence says	Implications of evidence	Claimed advantages	Claimed disadvantages	Resource requirements
Contacting study authors	6 studies	Identify: unpublished, or on-going studies, missing or incomplete data, completed but unpublished studies Expert in field to review includes	Contact original investigators through study report contact details—mainly e-mail/telephone	E-mail considered effective with better responses from institutional addresses	Additional studies identified; additional study data provided	No guarantee of additional or all relevant information identified Challenging and time consuming Less successful for older studies	Additional resources needed (may need up to 3 contact attempts with authors)
Citation chasing	9 studies	Identify: further studies, clusters or networks or studies	Backward and forward citation chasing using 3 electronic citation databases	Effectiveness of electronic citation methods unclear and suggest using all 3 databases	Not limited by keywords or indexing as bibliographic database searching is	Reliant on the currency, accuracy and completeness of the underlying citation network	Citation chasing of 46 studies = 79 h or 40 studies = 5 days
Handsearching	12 studies	Identify: studies or publications not routinely indexed in, or identified by, searches of bibliographic databases, including recently published studies	Manual examination of the contents of topic relevant journals, conference proceedings and abstracts	Use experts to develop list of journals to handsearch	Unique study identification, increased sensitivity; identifying studies missed or not indexed in databases	Studies still missed by handsearching; time and access to resources; low precision	Range between 6 min and 1 h per journal
Searching trial registers	3 studies	Identify: unpublished, recently completed or on-going trialsFind adaptations to trial protocols reported study outcomes	Comprehensive list of registries to search	Should be completed as complementary and not in isolation	Unique study identification	Search interfaces lag behind major databases	None reported
Web searching	5 studies	Identify: studies not indexed in bibliographic databases. Retrieving grey literature, study protocols and on-going studies	Relevant websites and using search engines	Use advanced search functions where possible	Unique study identification, hints to on-going or recently completed studies	Difficulties in transparent search, quality and quantity of searches returned	429 results in 21 h; google searching 7.9 h; targeted web searching 9-11 h

### Contacting study authors

The handbooks focus on identifying contact details and considering how to request studies or study data [9, 10, 13]. The studies evaluate the effectiveness of methods to make contact and elicit a response. Six empirical studies were included [6, 14–18].

#### What it is used for

It is used for identifying unpublished or on-going studies [10]; identifying missing, incomplete, discordant or unreported study data, or completed but unpublished studies [9, 13, 14, 16–18]; and asking study authors (or topic experts) to review a list of studies included at full text in a review, to see whether any studies had been inadvertently overlooked [9, 10].

#### What the evidence says

Two handbooks and one study provided detail on identifying contact details [6, 9, 10]. The Cochrane Handbook suggests that review authors should contact the original investigators, identifying contact details from study reports, recent publications, staff listings or a search of the internet [13]. Colleagues, relevant research organisations and specialist libraries can also be a valuable source of author information and contact details [9, 10]. A study by McManus et al. used a questionnaire, primarily to request study data or references, but also to ask recipients to recommend the names of other authors to contact [6]. A study by Hetherington et al. contacted authors and experts by letter in an attempt to identify unpublished trials [17].

Two studies reported using a multi-stage protocol to contact authors and request data: Selph et al. devised and followed a protocol that used both e-mail and telephone contact with the corresponding authors at defined stages over a period of 15 days [16]. Gibson et al. devised a similar protocol, although focused on e-mail contact, targeting first the corresponding authors and finally the last author and statisticians by e-mail and then telephone (statisticians were contacted due to the specific focus of the case study) [14]. Selph et al. contacted 45 authors and 28 (62%) provided study data [16], and Gibson et al. contacted 146 authors and 46 (31.5%) provided study data [14].

Two studies claimed that e-mail was considered an effective method of contact [14, 15]. O’Leary reported a response rate of 73% using e-mail contact, finding that more responses were obtained from an institutional address compared to a hotmail address (86 vs 57%, *p* = 0.02) [15]. Conversely, Reveiz et al. achieved a 7.5% response rate from contacting 525 study authors to identify RCTs but identified 10 unpublished RCTs and links to 21 unregistered and on-going RCTs [18]. Gibson et al. found that e-mail was most likely to receive a reply when compared to letter (hazard ratio [HR] = 2.5; 95% confidence interval [CI] = 1.3–4.0) but that a combined approach of letter and e-mail, whilst generating a higher response rate, was not statistically different from e-mail alone (73 vs 47%, *p* = 0.36. One hundred forty-six authors were contacted overall and 46 responded) [14].

Hetherington et al. sent letters to 42,000 obstetricians and paediatricians in 18 countries in an attempt to identify unpublished controlled trials in perinatal medicine [17]. Responses were received from 481 individuals indicating they would provide details concerning unpublished studies, and 453 questionnaires were completed and returned which identified 481 unpublished trials [17].

Chapter Seven of The Cochrane Handbook offers guidance on how to set out requests for studies or study data when contacting study authors [13]. The guidance suggests considering if the request is open-ended, or seeking specific information, and whether (therefore) to include a (uncompleted or partially completed) data collection form or request specific data (i.e. individual patient data) [13]. McManus et al. evaluated the use of a questionnaire to identify studies, study data and the names of relevant authors to contact for a systematic review [6]. The questionnaire resulted in the identification of 1057 references unique to the review, but no unpublished data were offered [6].

Two handbooks recommend submitting a list of included studies to authors [9] or topic experts [10] to identify any potentially missing studies. The Cochrane Handbook suggests including the review’s inclusion criteria as a guide to authors [9].

#### Claimed advantages

Five studies claimed that identifying additional published or unpublished studies, study data or references is possible by contacting study authors [6, 14, 16–18]. McManus et al. identified 23 references (out of 75 included in the review overall) by contacting study authors [6]; Reveiz et al. identified 10 unpublished RCTs and 21 unregistered or on-going RCTs [18]; two studies stated that they identified additional study data but did not separate their findings from contacting study authors from other methods of study identification [14, 16]; and Hetherington et al. identified 481 unpublished trials by contacting 42,000 obstetricians and paediatricians in 17 countries [17].

O’Leary found that more detailed study information was provided as a result of contacting study authors [14].

#### Claimed disadvantages

The CRD handbook claims that contacting authors/experts offers no guarantee of obtaining relevant information [10]. Selph et al. found that, whilst identifying additional studies or study data is possible, contacting study authors is challenging and, despite extensive effort, missing data remains likely [16].

Hetherington et al. claimed that methodologically sound trials were not reported through author contact, even by the investigators responsible for them. This was attributed, anecdotally, to the possibility that the trials yielded results that the investigators found disappointing [17].

Reveiz et al. reported low response rates. Of 525 study authors contacted, only 40 (7.5%) replied [18].

Two studies and one handbook claimed that contacting authors/experts is time consuming for researchers [10, 14, 16]. Selph et al. noted that this method is time consuming for the study authors too, who must identify the data requested [16].

Gibson et al. claimed that contacting authors/experts may be less successful for older studies, given the increased possibility that authors’ contact details are out of date [14]. Gibson et al. reported a 78% (CI = 0.107–0.479) reduction in the odds of response if the article was 10 years old or older [14].

#### Resource requirements

Gibson et al. claimed that additional resources were required to undertake author contact [14]. No specific details of the costs or time implications were recorded.

Gibson et al. recorded the duration between the information request and response [14]. This averaged 14 ± 22 days (median = 6 days) and was shortest for e-mail (3 ± 3 days; median = 1 day) compared to e-mail plus letter (13 ± 12 days; median = 9 days) and letter only (27 ± 30 days; median = 10 days) [14].

Selph et al. reported that all authors who provided data did so by the third attempt, suggesting that repeated attempts to elicit studies or study data may be ineffective [16].

### Citation chasing

The handbooks provide a brief overview of the method and list some of the tools commonly used [9, 10]. The studies typically evaluate the effectiveness of the tools used to undertake the search methods. Nine studies assessing the use of citation chasing were included [1–3, 19–24].

#### What it is used for

It is used for identifying further studies, and clusters or networks of studies, that cite or are cited by a primary study [10].

#### What the evidence says

Two studies provided detail on the application of the search method [1, 3]. The studies noted that backward citation searching is undertaken by reviewing bibliographies of relevant or included studies and forward citation chasing is undertaken by checking if a study, already known to be relevant, has since been cited by another study [1, 3].

Three tools for electronic citation searching dominate the studies: Web of Science, Scopus and Google Scholar. The first two are subscription databases, and Google Scholar is presently free [19].

#### Claimed advantages

Four studies claimed that an advantage of citation chasing is that it is not limited by keywords or indexing as is bibliographic database searching [2, 3, 20, 21]. Accordingly, four studies claimed the following advantages: Robinson et al. claimed that a small initial number of studies can create a network [21]; Hinde et al. claimed that citation searching can help inform researchers of parallel topics that may be missed by the focus of bibliographic database searches [2]; Janssens and Gwinn claimed that citation searching may be valuable in topic areas where there is no consistent terminology, so searches focus on links between studies rather than keywords [20]; and Papaioannou et al. reported that citation searching facilitated ‘serendipitous study identification’ due to the unstructured nature of citations [3].

One study appraised the quality of the studies identified through citation searching (and by other search methods) [3]. Papaioannou et al. reported that citation searching identified high-quality studies in their case study, although they do not define which quality appraisal tool was used to appraise study quality, so it is not clear if this observation is empirically derived [3].

#### Claimed disadvantages

Three studies stated that citation searching is reliant on the currency, accuracy and completeness of the underlying citation network [1, 21, 22]. Levay et al. identified ‘linking lag’, namely the delay between a study being cited and the citation being recorded in a citation database, which impacts on the currency of results [1]; Janssens and Gwinn stated that the accuracy and efficiency of citation searching depends on study authors citing studies, which means that selective citation of studies could cause relevant studies to be missed in citation searching [20]; Robinson et al. reported limited returns from citation searching where ‘broken citation links’ created ‘island’ studies which makes for incomplete citation networks and study identification [21].

Two studies questioned the efficiency of citation searching [2, 22]. Wright et al. screened 4161 studies to identify one study (yield rate of 0.0002) [22], and Hinde et al. screened 4529 citations to identify 76 relevant studies (yield rate of 0.0168) [2]. Wright et al. specifically recorded the time to undertake citation chasing in their study (discussed below in resource use), [22] whereas Hinde et al. did not report the time taken to search but state that the search was ‘very time consuming’ [2].

Two studies claimed that replicability of citation searching strategies could be affected by the choice of the tools used [1, 24]. Levay et al. questioned the replicability of Google Scholar, since search returns are controlled by Google’s algorithm, meaning that the results returned will change over time and cannot be replicated [1]. Bramer et al. found reproducibility of citation searching to be low, due to inaccurate or incomplete reporting of citation search strategies by study authors [24].

#### Resource requirements

Two studies recorded the time taken to citation search, and one study commented on the time needed [1, 3, 22]. Levay et al. reported that citation searching the same 46 studies in Web of Science and Google Scholar took 79 h (Web of Science = 4 h and Google Scholar 75 h) to identify and de-duplicate 783 studies (Web of Science = 46 studies and Google Scholar = 737 studies) [1]. Wright et al. reported that citation chasing the same 40 studies in Web of Science, Medline, Google Scholar and Scopus took 5 days in total (2 days to download 1680 results from Google Scholar; 1 day to download 2481 results from Web of Science, Scopus and Medline; and 2 days to screen all the studies) [22]. Both studies commented on the administrative burden of exporting studies from Google Scholar which accounted for the majority of time searching in both cases [1, 22]. Conversely, Papaioannou et al. claimed reference tracking and citation searching to be minimally time intensive, yielding unique and high-quality studies. The number of studies citation chased, the time taken to search and the tool used to appraise study quality were not reported [3].

One study provided data on the costs involved in citation chasing [1]. Levay et al. reported that the staff time to search Web of Science for 4 h cost between £88 and £136 and the 75 h to search Google Scholar cost between £1650 and £2550, based on staff grades ranging from £22–£34 per hour (all UK Sterling: 2012) [1].

### Handsearching

The handbooks focus on where to handsearch [9, 10], and they provide guidance on who should do this [9]. The studies have a similar focus but they have sought to evaluate effectiveness compared with other search methods [25–28] as well as to evaluate the effectiveness and/or the efficiency of handsearchers in identifying studies [29, 30]. Twelve studies were included [25–36].

#### What it is used for

It is used for ensuring the complete identification of studies or publication types that are not routinely indexed in, or identified by, searches of bibliographic databases, including recently published studies [10].

#### What the evidence says

Handsearching involves a manual, page-by-page, examination of the entire contents of relevant journals, conference proceedings and abstracts [9, 10, 27, 31].

Two handbooks and six studies provide detail on selecting journals to handsearch [9, 10, 25, 27, 30–33]. Three strategies were identified, as set out below.

##### Using databases (or database search results) to identify journals to handsearch

The handbooks suggest that bibliographic databases can be used to identify which journals to handsearch [9, 10]. The Cochrane Handbook, with its focus on identifying studies reporting randomised controlled trails (RCTs), suggests that searches of The Cochrane CENTRAL database, MEDLINE and EMBASE can be used to identify journals that return the greatest number of studies by study design in the relevant topic area of research [9]. Variations of this approach to selecting journals to handsearch were utilised in three studies [25, 30, 31]. The CRD Handbook suggests analysing the relevant results of the review’s bibliographic database searches in order to identify journals that contain the largest number of relevant studies [10].

##### Handsearching journals not indexed in bibliographic databases

The Cochrane Handbook suggests that journals not indexed in MEDLINE or EMBASE should be considered for handsearching [9]. A study by Blümle et al. considered this strategy necessary to obtain a complete search [32].

##### Contacting experts to identify journals to handsearch

Two studies contacted experts to develop a list of journals to handsearch [30, 31]. Armstrong et al. contacted organisations to develop a list of non-indexed journals to handsearch (in addition to database searching), and Langham et al. used a combination of database searches, contacting organisations and searches of library shelves to identity relevant journals (in addition to database searching) [30, 31]. A list of possible journals to handsearch was provided to professional contacts to appraise and identify any missing journals [30]. Neither study specifically reports the number of journals identified by experts to handsearch, when compared to the number of journals to handsearch identified by database searching, and there is no discussion of the effectiveness of either method in identifying journals to handsearch.

Five studies explored specifically where or which sections of a journal to handsearch [25, 27, 28, 31, 33]. A study by Hopewell et al. handsearched full reports, short reports, editorials, correspondence sections, meeting abstracts and supplements [27]. Hopewell et al. found that, of the 369 reports uniquely identified by handsearching, 92% were abstracts and/or published in the supplement of journals [27]; two studies reported the greatest value in searching supplement editions of journals [28, 31], since these are not routinely indexed in databases [28]. Armstrong et al. identified three studies (out of 131) through searching supplement editions of journals [31], and Jadad et al. identified 162 eligible RCTs from a total of 2889 abstracts reported in four journals [28]; Croft et al. claimed value in searching the correspondence section of journals but they did not record the effect of handsearching this section in terms of identification of studies [33]; and Adams et al. reported handsearching book reviews and identifying one study [25].

#### Claimed advantages

Table 3 summarises a claimed advantage of handsearching, since the studies demonstrate that handsearching identifies studies missed through database searching. Where the studies reported the reason that the studies were missed by database searching (the advantage of handsearching), these are summarised in Table 3.

**Table 3:** Handsearching results

	Advantages		Disadvantages	
Studies	No. identified by handsearching but missed by MEDLINE	Why studies were missed by MEDLINE (claimed advantages of handsearching)	No. identified by MEDLINE but missed by handsearching	Why studies were missed by handsearching (claimed advantages of database searching)
Adams et al. [25]	9% (67 out of 698) of RCTs (CI 7–11%; Sensitivity 94% (CI 93–95%);Precision 7% (CI 6–8%).	▪ Conference abstracts and letters not indexed in databases; ▪ RCTs not indexed, or no methodological data available to identify studies; ▪ Methodological descriptors (i.e. ‘random’ for allocation) were overlooked by database indexers.	*Standard MEDLINE search*: sensitivity 18% (CI 15–21%) Precision 40% (CI 35–45%) *Skilled MEDLINE searched:* Sensitive: 52% (CI 48–56%) Precision 59% (CI 55–65%)	▪ Studies missed by searcher error/fatigue; ▪ Methodological data being ‘hidden’ in article
Armstrong et al. [31]	6 out of 131 (4.6%) RCTs/CCTs	▪ Trials made no reference in abstract, title or subject headings to random allocation; ▪ Trials used terms for random allocation it the title, abstract or MeSH but were not correctly indexed by publication type; ▪ Trials were abstracts; ▪ Studies were identified in supplement editions of journals not indexed in MEDLINE; and ▪ Not found in MEDLINE as issue appeared missing in MEDLINE.	125 (of 131) studies *would* have been identified by a MEDLINE using PICO search. 118 (of 131) *would* have been identified by a PICOs search	Not reported
Blümle and Antes[32]	10, 165 RCTs/CCTs out of 18,491(55%)	▪ Incorrect indexing and incomplete compilation of health care journals in electronic databases impair result of systematic literature search.	Not reported in abstract	Not reported in abstract
Croft et al. [33]	7 out of 10 (70%)	▪ Two RCTs identified through letter to editors ▪ Not picked up in MEDLINE search	3 studies identified in MEDLINE (30%)	Not reported
Glanville et al. [26]	7 out of 25, although none of these studies met the review’s inclusion criteria.	Not reported	Electronic searching (including reference checking), by comparison, yielded 30 included papers.	Not reported
Hay et al. [26]	5 of 40 studies identified (compared to EMBASE) or 13 of 40 (compared to PsycLIT)	Not reported	EMBASE *n* = 35 (out of 40) RCTs (88%) and precision 9%. PsycLIT *n* = 27 (out of 40) and precision 9%.	EMBASE: ▪ *n* = 3 journal years not indexed ▪ *n* = 2 reason unclear. PsycLIT: ▪ *n* = 13 gap in indexing and current material being loaded.
Hopewell et al. [27]	369 out of 714 (52%) RCTs	▪ 252/369 (68%) no MEDLINE record. ▪ 232/252 (92%) abstracts and/or published in supplements.	32 of 714 (4%)	
Jadad et al. [28]	*Handsearching* vs *MEDLINE* 25 out of 151 (16.5%)precision 2.7%*handsearching* vs *supplement editions and MEDLINE.* 150 out of 162 eligible (precision 5.6%).	▪ Non-indexed abstract (n = 7); ▪ Non-indexed letter (*n* = 1); ▪ Search term random not in MeSH or abstract summary (n = 9); ▪ Key search term not in MeSH or in abstract summary (*n* = 7); and ▪ No apparent reason (n = 1).	*handsearching* vs *MEDLINE* 2 of 245 (0.8%) *handsearching* vs *supplement editions and MEDLINE.* 1 out of 13 (7.6%)	▪ Why studies were missed by handsearching is not reported or explored
Langham et al. [30]	227 out of 710 (32%)	Not reported	MEDLINE identified 118 (16.6%) of studies missed by Handsearching.	Not reported
Mattioli et al. [35]	0 out of 25 (0), (all identified by handsearching)	Not reported	*Specific PubMed Search* 16 out of 25 (64%) *Sensitive PubMed Search* 9 out of 25 (36%)	Not reported
Milne and Thorogood [36]	34 out of 82 (41.5%)	Not reported	Capture/recapture used to test. Estimated *n* = 3 missed by handsearching.	▪ Inadequate indexing or trials not indexed on MEDLINE ▪ Prohibits are not located by computerised searches

#### Claimed disadvantages

Table 3 also summarises a claimed disadvantage of handsearching since, even though this method is often defined as a ‘gold standard’, the studies demonstrate that database searching can identify studies missed by handsearching. Where the studies reported the reason that the studies were missed by handsearching (the disadvantage over database searching), these are summarised in Table 3.

Two studies claimed that the precision of handsearching was low when compared to the precision found in database searching [25, 28]. Table 3 records the relative precision between handsearching and MEDLINE searching. Two studies claimed that the time needed to handsearch, and access to resources (including handsearchers), was a disadvantage of handsearching [31, 36].

#### Resource requirements

Seven studies reported detail on the time taken to handsearch [25, 28, 29, 31, 33, 34, 36]. There was no agreement between the studies on how long handsearching takes. The range was between 6 min [36] and 1 h [29] per journal handsearched. It is not possible to calculate an average, since not all studies reported their handsearching as time per journal handsearched. One study reported handsearching in ‘two hour bursts’ across 3 months in order to focus concentration, but the detail of how often these ‘bursts’ occurred and the effectiveness relative to ‘non-burst’ handsearching is not reported [33].

Jadad et al. reported the time taken specifically to handsearch the supplement editions [28]. Two thousand, eight hundred and eighty-nine abstracts were handsearched in 172 min with an average of 1.1 min per eligible study identified [28].

The use of volunteers [29, 30] or experienced handsearchers [27, 31] varied in studies. Due to the varied outcome measures used between the studies, it is not possible to aggregate the effectiveness of experienced handsearchers against volunteers. Moher et al., however, specifically sought to test the effectiveness of volunteers in identifying RCTs, finding that volunteers with minimal training can contribute to handsearching [29]. Conversely, a study by Langham et al. discussed a possible explanation of their volunteer handsearcher missing studies was a lack of specific knowledge to identify RCTs [30], which suggests experience or training is necessary. Milne and Thorogood suggested that handsearching may need to be undertaken by more than one person [36].

Five studies provided data on training given to handsearchers [25, 27, 29, 30, 34]. This included specific training on RCTs [27, 29], a 2-h training session [29, 34] and an information pack including guidelines to handsearching, developed by experienced handsearchers, and a thesaurus of terms to identify RCTs [30].This data was reported narratively, and supporting information, such as the information pack reported in the study by Langham et al., was not provided in the studies. [30].

Two studies provided guidance on approaches to handsearching if resources were limited [27, 28]. Hopewell et al. claimed that, where resources are limited (and it was accepted that studies would be missed), and the aim of searching is the comprehensive identification of studies reporting RCTs, handsearching is best targeted on journals not indexed in MEDLINE and journals published before 1991 (the year the publication type indexing term for RCTs was introduced into MEDLINE [37]) [27]. Jadad et al., in a study focused on identifying RCTs, claimed that a combination of MEDLINE searches with selective handsearching of abstracts of letters may be a good alternative to comprehensive handsearching [28].

Armstrong et al. claimed that researchers handsearching for non-randomised study designs may need more time to handsearch. No guidance on speculative timing was given [31].

Moher et al. provided data on costs. Moher et al. recorded costs for photocopying (10–15 Cents Canadian per page) and car parking (10 Dollars Canadian) in their 1995 study assessing the use of volunteers to handsearch [29].

### Searching trial registers

The handbooks focus on the benefit of searching registers [10], with The Cochrane Handbook providing specific guidance on where to search [9]. The studies focused on the searching of the registers [38] and the advantages and disadvantages of doing so [39, 40]. Three studies were included [38–40].

#### What it is used for

It is used for identifying unpublished, recently completed or on-going trials [9, 10, 39, 40] and keeping a track of any adaptations to trial protocols and reported study outcomes [39, 40]. Trials that have been stopped, or were unable to reach optimal recruitment, can also be identified.

#### What the evidence says

The Cochrane Handbook includes a comprehensive list of trial registers to search [9]. Distinctions are made between national and international trial registers (which hold trials of any population or intervention), subject (i.e. population)-specific registers and pharmaceutical/industry trial registers [9]. There is a further distinction between on-going, completed trial registers and result registers. Glanville et al. also drew a distinction between trial registers (e.g. ClinicalTrials.gov) and portals to trial registers (e.g. WHO) [38].

Glanville et al. explored the need to search trial registers as a complementary search method to comprehensive searches of bibliographic databases [38]. Glanville et al. reported that, in both ClinicalTrials.gov and WHO International Clinical Trials Registry Platform (ICTRP), their ‘highly sensitive single concept search’ of the basic interface offered the greatest reliability in identifying known records. The methods of searching are explored in greater detail in this study [38].

#### Claimed advantages

Two studies claimed that searching trial registers will identify unique studies or study data [39, 40]. Van est. et al. reported that, in four out of 80 Cochrane reviews included in their study, primary studies were identified and included from a prospective search of a trial register search [39]. Jones et al. reported that, of 29 studies to record registry search results in their study, 15 found at least one relevant study through searching a register [40].

Two studies claimed that searching of trial registers facilitates checking of a priori outcome measures against reported final outcome measures [39, 40]. Jones et al. suggested that the comparison of registered trials (and trial data) against published trials (and data) will aid the understanding of any potential bias in the trials [40].

Jones et al. noted that an advantage of trial registers is that they often include contact details for trial investigators, thereby facilitating author contact [40].

#### Claimed disadvantages

Two studies concluded that trial registers must be searched in combination with other bibliographic resources [38, 39]. Glanville et al. concluded that trial registers lag behind major bibliographic databases in terms of their search interfaces [38].

#### Resource requirements

None were reported.

### Web searching

The handbooks report limited guidance for web searching. The CRD Handbook suggests that web searching may be a useful means of identifying grey literature [10], and The Campbell Handbook provides some guidance on how to undertake web searches, including a list of grey literature websites [11]. The studies explored the role of web searching in systematic reviews. Five studies were included [41–45].

#### What is it used for

It is used for identifying published or unpublished studies not indexed or included in bibliographic databases, or studies missed by database (or other) search methods, identifying and retrieving grey literature and identifying study protocols and on-going studies [10, 11, 42, 45].

#### What the evidence says

The CRD Handbook makes a separation between a search of the internet through a ‘search engine’ and searches of specific and relevant websites [10]. It considers the latter to be more practical than a general search of the World Wide Web in systematic reviews [10].

The Campbell Handbook provides guidance on searching using a search engine [11], and Eysenbach et al. reported the results of a pilot study to assess the search features of 11 search engines for use in searching for systematic reviews [45].^1^ The Campbell Handbook suggests that, when using search engines, researchers should use the advanced search function. In some cases, this allows searchers to use Boolean logic and employ strategies to limit searches, such as precise phrases like “control group” [11].

Godin et al. reported the development and use of a web-searching protocol to identify grey literature as part of study identification in a systematic review [43]. Godin et al. broke their web searching into three parts: first, searches using Google for documents published on the internet; secondly, searches using custom Google search engines; and thirdly, browsing targeted websites of relevant organisations and agencies [43].

#### Claimed advantages

Two studies identified studies uniquely by web searching [43, 45]. Eysenbach et al. identified 14 unpublished, on-going or recently finished trials, and at least nine were considered relevant for four systematic reviews [45]. Godin et al. identified 302 potentially relevant reports of which 15 were included in their systematic review [43].

Three studies commented on the types of study or study data identified [42, 43, 45]. Eysenbach et al. claimed that internet searches may identify ‘hints’ to on-going or recently completed studies via grey literature [45]; Godin et al. uniquely identified report literature [43]; and Stansfield et al. suggested that web searching may identify studies not identified from ‘traditional’ database searches [42].

#### Claimed disadvantages

Five studies discussed the disadvantages of web searching [41–45]. The studies drew illustrative comparisons between database searching and web searching in order to highlight the disadvantages of web searching:

Three studies commented on searching using a web search engine: Eysenbach et al. reported that current search engines are limited by functionality and that they cover only a fraction of the visible web [45]; Mahood et al. claimed that their chosen search engines could not accommodate either full or modified search strategies, nor did they support controlled indexing [44]; and Godin et al. claimed that, in contrast to systematic searches of bibliographic databases, where one search strategy combining all search terms would be used, Google searches may require several search enquiries containing multiple combinations of search terms [43].

Three studies commented on the number of studies returned through web searching [43–45]. Godin et al. claimed that searching Google can be overwhelming due to the amount of information and lack of consistent organisation of websites [43]; Mahood et al. had to limit their web searches to title only in order to control search returns [44], and Eysenbach et al. recorded recall of between 0 and 43.6%, finding references to published studies and precision for hints to published or unpublished studies ranged between 0 and 20.2% [45].

Three studies commented on the search returns [42, 43, 45]. Eysenbach et al. and Stansfield et al. commented on the lack of abstracts when web searching, which impacts on the precision of web searching and volume of studies identified [42, 45], and Godin et al. claimed that it was impossible to screen all results from a Google search, so researchers were reliant on page ranking [43].

Three studies claimed potential issues with the reliability of items identified through web searching [41, 43, 45]. Godin et al. discussed the possibility of bias created in web searching, where search results are presented depending on geographic location or previous search history [43]; Briscoe reported that algorithms used by search engines change over time and according to the user, which will influence the identification of studies and impact the transparency and replicability of search reporting [41]; and Eysenbach et al. reported identifying a study published on-line that differed in reporting to the copy published in the peer-reviewed journal, where adverse event data was omitted in the on-line version [45].

Stansfield et al. claimed that the lack of functionality to export search results presented a challenge to web searchers [42]. Three studies claimed that web searching presented difficulties in transparent search reporting [41, 43, 44].

#### Resource requirements

Two studies discussed time taken to web-search [43, 45]. Eysenbach et al. reported searching 429 returned search result pages in 21 h [45], and Godin et al. reports custom Google searching taking 7.9 h and targeted web searches taking 9–11 h, both timings being specific to the case studies in question [43].

Stansfield et al. discussed planning when to undertake web searching [42]. Stansfield et al. linked planning a web search to the time-frame and resources available in order to inform where to search [42].

Mahood et al. claimed that large yields of studies can be difficult and time consuming to explore, sort, manage and process for inclusion [44]. Mahood et al. initially had to limit their web searching to title only (as a method to control volume) before eventually rejecting their web searching due to concerns about reproducibility and ability to manage search returns [44].

No studies reported any data relating to the costs involved in web searching.

## Discussion

The discussion will focus on two elements inherent in the research question of this study: how does current supplementary search practice compare with recommended best practice and what are the implications of the evidence for searching using these supplementary methods.

### Contacting study authors

The advent of e-mail (and more specifically the standardised reporting of e-mail addresses for corresponding study authors) would appear to have improved the efficiency of contacting study authors [10, 11], although it is possible that it has not altered the effectiveness [46]. Identifying additional studies or data (the effectiveness) is conditional upon a reply, whatever the method of contact. The guidance of the handbooks, to consider how best to set out requests for studies or study data, is well made but seldom explored in the studies themselves. Whilst making contact is important, which the studies evaluate exploring techniques to improve the rate of reply would be a valuable contribution to improve the efficiency and effectiveness of identifying studies or study data through author contact.

When to contact study authors is worthy of consideration, since the studies included in this review reported a delay between asking for studies or study data and a response. Sufficient time should be allowed between identifying the need for author contact, making contact, a response being provided and the study or data being integrated into the review (with all the methodological implications considered). A recognition for the need of this method, combined with the realisation that this method takes time to yield results, is important. It is perhaps for this reason that, whilst contacting authors is common in systematic reviews, it is not a method of study identification that is undertaken as a matter of course [47].

The concept of contacting authors could also be understood more broadly than simply contacting with a view to requesting known studies or data. Whilst in contact with authors, requests for unpublished, linked or forthcoming studies are not unreasonable requests, and authors can assist with the interpretation of specific elements of studies or topics, in order to aid the process of critical appraisal. Furthermore, Ogilvie et al. found the value in contacting experts was the link to better reports of studies already identified [48]. This highlights the potential flexibility of the search method: it is not only the chance to identify known studies or study data but also it offers the opportunity to *speak* with experts.

### Citation chasing

The advantages and disadvantages (and resource requirements) were most clearly stated for this supplementary search method. The handbooks, and some studies, suggested and found advantages and disadvantages in the methods and tools.

The Cochrane Handbook suggested that there is little evidence to support the methodology of citation searching, since the citation of studies ‘is far from objective’ [49]. The studies included in this review suggested that the reasons for ‘non-citation’ are unclear and could range from selective citation (i.e. selective reporting) to pragmatic reasons, such as a review of trials being cited instead of each individual trial reviewed [21]. Furthermore, a high number of citations for a study should not necessarily be confused as an indicator of study quality [50, 51] or a complete citation network. Non-citation of studies, or ‘linking lag’ [1], forces a break in citational networks [1, 2, 21], meaning it becomes unclear when (or if all) studies have cited a primary study [20]. There is presently no method to assess the completeness of citational networks and no certainty as to the comprehension of any citation chasing.

There is little common agreement between the studies as to which tool (or combination of tools) is superior in citation chasing, since the relative merits of each resource depend greatly upon the topic of review, the data range of the resource and the currency of the results (c.f. [1, 23, 24, 52–54]). A study that evaluated the tools (Web of Science, SCOPUS and Google Scholar), how the tools are best searched, how the platform hosts select data for inclusion and the advantages and disadvantages of use would make clearer statements on when (or if) to use which tools.

There are, undoubtedly, advantages to citation searching. The citational link is neutral, in the sense that it only links the studies but it does not explain the nature of the link. This is important, since a citation search will identify *any* study linked to the primary study, including erratum studies and studies that dispute or disagree with the primary study, and it should also link different publication types, such as editorial content, reviews or grey literature. This could not only aid interpretation of studies but also it could help researchers explore the idea of study impact. Furthermore, as reported in the ‘Results’ section, a citation search links by citation and it is not beholden to the use of ‘the correct’ search terms or database indexing. It may, therefore, as Papaioannou et al. reported, facilitate serendipitous study identification [3], suggesting that citation chasing is valuable in scoping review topics, to aid development of searches, and review searches, in order to ensure all studies have been identified.

The nature of bi-directional citation chasing suggests that, given the relative specificity, this method could possibly be used to efficiently update systematic reviews using known includes as the citations to chase [20]. Researchers have had positive, although incomplete, success trialling this method, and studies suggest that citation chasing alone is not a substitute for standard update searches [55, 56].

### Handsearching

The evidence on handsearching can be summarised as (1) selecting where to handsearch, (2) what to handsearch and (3) who does the handsearching. In relation to 1, the handbooks advocate selecting journals to handsearch on the basis of the number of relevant studies included from journals identified in database searching. This approach means handsearching is a supplementary method to database searching, since to undertake handsearching—following this method—database searches define the list of journals to handsearch.

Studies included in this review provided empirical evidence that handsearching journals identified by database searching was effective in identifying studies missed by poor indexing, lack of study design or omission of key search terms, or where sections of journals are not indexed on databases. In this way, this approach to selecting journals to handsearch could be categorised as a ‘safety net search’, since it aims to identify studies missed by deficiencies in literature searching and database indexing. This approach to selecting journals to handsearch, even though it is effective, could be argued to be a duplication of effort, since the journals being handsearched have already been ‘searched’ through the bibliographic databases. This is likely why the studies recorded low precision (compared to database searches) and why handsearching takes longer [28].

The Cochrane Handbook and three studies suggested alternative ways to identify journals to handsearch: namely, selecting journals not indexed on MEDLINE or EMBASE [9, 32]—a suggestion that is easily changed to read ‘primary databases’ relevant to the field of study (i.e. ERIC for reviews of educational topics)—and contacting experts, contacting organisations and searches of library shelves [30, 31]. Neither the study by Armstrong et al. nor the study by Langham et al. listed the journals identified by method of identification, so it is not clear if there were differences between the list of journals provided by experts when compared to those provided by databases [30, 31]. This review did not identify any studies that compared the use of databases to identify journals to handsearch as against these alternative methods but such a study may be of value if efficiencies could be found in practice.

It may be that, in reviews in which a comprehensive identification of studies is required, identifying journals to handsearch should be done both by using databases and contacting experts or organisations. The former being to cover any deficiencies in the database searching and the latter to capture any unique journals or conferences known to experts but not indexed in databases.

Selecting what to handsearch and who should handsearch was another notable difference between the handbooks and studies. The studies included in this review identified studies uniquely from handsearching various sections of journals (from abstracts through to book reviews), and the studies used volunteers, provided training to handsearchers, and used experienced handsearchers to handsearch, with varying degrees of success and failure since handsearching relates to effectively identifying studies when compared to database searching. The Cochrane Collaboration arguably has one of the longest track-records of handsearching projects (c/f [37]), and it is their recommendation that handsearching is the page-by-page examination of the entire contents of a journal [9, 10] by a well-trained handsearcher [9]. Handsearching is commonly referred to and used as a ‘gold standard’ comparator to establish effectiveness of other search methods. Given that every study included in this review uniquely identified studies by handsearching but also missed studies by handsearching too, a reminder of what constitutes handsearching is likely warranted.

### Trial registers

The handbooks provide guidance on where to search and the studies focused on the effectiveness of study identification in selected registers and/or the practicalities of searching registers. In this way, the studies advance the guidance of the handbooks, since they provide empirically derived case-studies of searching the registers. The implications for searching, however, are clear: searching trial registers should still be undertaken in combination with bibliographic database searching [38, 57]. Even despite the aims of the International Committee of Medical Journal Editors [58], comprehensive and prospective registration of trials—and keeping the trial data up to date—is still not common place. It is unclear what pressure (if any) is put upon trial managers who do not prospectively register their trials and, in fact, if there is any active penalty if trial managers do not do so. Until this issue is resolved, the comprehension of registers will remain uncertain and a combination of bibliographic database searching (to identify published trials) and searches of trial registers (to identify recruiting, on-going or completed trials) is required.

The advantages of searching trial registers are worthy of discussion. Registered trials include an e-mail address for trial managers, which can facilitate author contact, and the studies concluded that more consistent searching of trial registers may improve identification of publication and outcome reporting bias [40, 59]. If trial managers were using the portals correctly, it would also be a practical method of reporting results and sharing study data, perhaps akin to a ‘project website’, as recommend in the Cochrane Handbook [9]. The variability of the search interfaces is notably a disadvantage and something upon which could be improved. Glanville et al. observed that the search interfaces lag behind major bibliographic databases [38]. If the registers themselves are hard to search (and in some cases impossible to export data from), they are less likely to be searched. Trial managers and information specialists/researchers could usefully work together with the registers to develop the interfaces in order to meet the needs of all who use them. The use of trial registers may be broader than only researchers [60].

### Web searching

In their 2001 study, Eysenbach et al. stated that the role of the internet for identifying studies for systematic reviews is less clear when compared to other methods of study identification [45]. The handbooks do not update this view, and very few studies were identified in this review which improve upon Eysenbach et al.’s claim. The studies have attempted to take on Eysenbach et al.’s suggestion that a systematic investigation to evaluate the usefulness of the internet for locating evidence is needed. Mahood et al., however, had to abandon their attempts to web-search [44], but Godin et al. took this work a little further in their case study with reference to identifying grey literature [43].

The comparative lack of guidance in the handbooks could stem either from a lack of certain knowledge of how to web-search or perhaps a lack of certainty of how to do this systematically, such that web searching could be replicable, and therefore, be included as a method to identify studies without introducing bias. Researchers are exploring the idea of how far web searching can meet the need to be replicable and transparent but still functional [41]. Further guidance is undoubtedly needed on this supplementary search method.

### Limitations

The date range and age of the handbooks and studies included in this review could be considered a limitation of this study.

Comparative and non-comparative case studies form the evidence base for this study. The studies included in this review have been taken at face-value, and no formal quality appraisal has been undertaken since no suitable tool exists. Furthermore, supplementary search methods are typically evaluated in the context of effectiveness, which is potentially a limited test of the contribution they may offer in the process of study identification. Different thresholds of effectiveness and efficiency may apply in the use of supplementary search methods in systematic reviews of qualitative studies when compared to reviews of RCTs, for example.

The studies themselves do not necessarily correlate to the concepts of claimed advantages and disadvantages. In most cases, proposed advantages and disadvantages have not been tested in practice.

Whilst we have aimed to comprehensively identify and review studies for inclusion, the use of supplementary search methods is a broad field of study and it is possible that some completed studies may have been inadvertently missed or overlooked. It is possible that standard systematic review techniques, such as double-screening, would have minimised this risk, but we are confident that, whilst a more systematic approach may have improved the rigour of the study, it is unlikely to alter the conclusions below.

## Conclusions

Current supplementary search practice aligns methodologically with recommended best practice. The search methods as recommended in the handbooks are perceptibly the same methods as used in the studies identified in this review. The difference between the handbooks and the studies is of purpose: the studies sought to test the search methods or tools used to undertake the search methods.

The causal inference between methods (as presented in the handbooks) and results (as found in the studies) could be usefully tested to develop our understanding of these supplementary search methods. Further research is needed to better understand these search methods. Specifically, consistency in measuring outcomes, so the results can be generalised and trends identified, which would provide a link not only to better effectiveness data but also to efficiency data, offers researchers a better understanding of the value of using these search methods, or not.

### Time

All of the studies discussed in this review claimed to identify additional includable material for their reviews using supplementary search methods that would have been missed using database searches alone. Few of the studies, however, reported the resources required to identify these unique studies. Further, none of the studies used a common framework or provided information that allows a common metric to be calculated. It is not, therefore, possible to compare the resources required to identify any extra study with each search method. This, alongside the use of comparative and non-comparative case studies as the primary study design to test effectiveness, limits our ability to generalise the results of the studies and so reliably interpret the broader efficiency of these search methods. Researchers could usefully consider reporting the amount of time taken to undertake each search method in their search reporting [28, 61].

### Value versus impact?

Identifying unique studies is commonly interpreted as adding value to the review and the process of searching in and of itself. Only three studies sought to extend this, appraising either the quality of the studies identified or the contribution of the studies to the synthesis as a way of considering the value of the additional studies [3, 16, 45]. In reviews of effectiveness, where all studies should be identified so as to generate a reliable estimate of effect, study value might be a moot point but, in resource-limited situations, or for reviews where a comprehensive identification of studies is less important, study value is an important metric in understanding the contribution of supplementary search methods and the extent to which researchers invest time in undertaking them.

### Time + value

Comparing the time taken to search, with a summary estimate of the contribution or value of the studies identified uniquely, against the total number of studies identified, could alter how researchers value supplementary searches. It would permit some basic form of retrospective cost-effectiveness analysis, which would ultimately move literature searching beyond simply claiming that more studies were identified to explaining what studies were identified, at what cost and to what value.

## Abbreviations

RCT: Randomised controlled trial

## Footnotes

1 Eysenbach et al. recommend Alta Vista but this search engine no longer exists.
